# A Role for Exchange of Extracellular Vesicles in Porcine Spermatogonial Co-Culture

**DOI:** 10.3390/ijms23094535

**Published:** 2022-04-20

**Authors:** Shiama Thiageswaran, Heather Steele, Anna Laura Voigt, Ina Dobrinski

**Affiliations:** 1 Department of Biochemistry and Molecular Biology, Cumming School of Medicine, University of Calgary, Calgary, AB T2N 4N1, Canada; shiama.thiageswaran@ucalgary.ca; 2 Department of Comparative Biology and Experimental Medicine, Faculty of Veterinary Medicine, University of Calgary, Calgary, AB T2N 1N4, Canada; heather.steele@ucalgary.ca (H.S.); anna.voigt1@ucalgary.ca (A.L.V.)

**Keywords:** spermatogonial stem cells, spermatogonia, co-cultures, extracellular vesicles, exosomes, pig

## Abstract

Spermatogonial stem cells (SSCs) provide the basis for lifelong male fertility through self-renewal and differentiation. Prepubertal male cancer patients may be rendered infertile by gonadotoxic chemotherapy and, unlike sexually mature men, cannot store sperm. Alternatively, testicular biopsies taken prior to treatment may be used to restore fertility in adulthood. Testicular SSC populations are limited, and in vitro culture systems are required to increase numbers of SSCs for treatment, demanding culture systems for SSC propagation. Using the pig as a non-rodent model, we developed culture systems to expand spermatogonia from immature testis tissue, comparing different feeders (Sertoli cells, peritubular myoid cells (PMCs) and pig fetal fibroblasts (PFFs)). Spermatogonia co-cultured with Sertoli cells, PMCs and PFFs had comparable rates of proliferation and apoptosis. To elucidate the mechanism behind the beneficial nature of feeder layers, we investigated the role of extracellular vesicles in crosstalk between spermatogonia and feeder cells. Sertoli cell-released exosomes are incorporated by spermatogonia, and inhibition of exosomal release reduces spermatogonial proliferation. Together, these results show that PMCs, PFFs and Sertoli cells promote spermatogonial proliferation in co-culture, with exosomal exchange representing one possible mechanism. Further characterization of exosomal cargo may ultimately allow the development of feeder-free culture systems for clinical use.

## 1. Introduction

Spermatogonial stem cells (SSCs) are the basis of male fertility. SSCs reside in the SSC niche, along the basement membrane of the seminiferous tubules [1,2,3,4]. Interactions in the niche support the maintenance of SSC self-renewal and differentiation [2,5,6]. SSCs may be utilised to preserve male fertility in individuals in which sperm preservation is not an option [7,8]. This is of particular importance to pre-pubertal male cancer patients that are rendered potentially infertile by gonadotoxic chemotherapy treatment. Autologous transplantation of SSCs after isolation and in vitro culture may restore the patient’s fertility in adulthood.

SSCs make up a small population of cells within the testis [9] and a specific marker to select and enrich populations of SSCs has not yet been identified [10]. Therefore, the quantity of cells required for effective experimentation and therapy development can only be acquired by increasing the numbers of spermatogonia, which contain SSCs, in vitro [11]. However, the establishment of functional culture systems remain to be sufficiently developed in species other than rodents and closely related smaller species [12,13,14,15,16].

A culture system to support the growth and proliferation of murine spermatogonia was first established using mouse fibroblast feeder cells and media supplemented with GDNF, FGF2, EGF, and LIF [12] before the development of feeder-free culture conditions [17,18,19,20], and translational studies to culture spermatogonia from more closely related species including hamsters, rats, and rabbits [13,15,16,18,21]. However, SSCs from larger animals and humans cannot be similarly supported by these established conditions, possibly due to differences in metabolic requirements during the extended prepubertal developmental phase [22,23,24].

In a previous study, neonatal Sertoli cells were the most supportive feeder cells for prepuberal porcine spermatogonia [25]. However, this study compared porcine Sertoli cells (neonatal and adult) against Sandos inbred mice (SIM) 6-thioguanine-resistant, ouabain-resistant (STO) cells [25] and did not explore other cell types found in the testicular niche. Lee et al., 2016 found that neonatal porcine germ cell-derived colonies could be sub-cultured on testicular somatic cells expressing GATA4 [26], indicating that they may also be suitable feeder layers in co-culture conditions. Within the niche, SSCs reside in proximity with Sertoli cells and other testicular somatic cells such as peritubular myoid cells (PMCs) and testicular endothelial cells (TECs), which secrete glial cell-derived neurotrophic factor (GDNF) and other factors that promote SSC proliferation and the maintenance of stemness [27,28,29,30,31,32].

Aside from providing growth factors and nutrients, the benefits of co-culture may also be mediated through the exchange of extracellular vesicles (EVs), such as microvesicles and exosomes. Microvesicles and exosomes range in diameter from 100 nm–1 µm and 30–150 nm, respectively, and are released from most cell types [33,34,35]. Both microvesicles and exosomes contain a variety of biologically active cargo such as proteins and nucleic acids, and are involved in intercellular communication, as the uptake of these secreted vesicles can yield changes in recipient cell physiology [36,37,38]. It is possible that the close contact between feeders and cultivated cells in co-culture conditions facilitates exosomal exchange between the two populations, whereby spermatogonia receive beneficial factors for proliferation and survival, and feeders receive feedback from spermatogonia to inform future cargo sorting.

Therefore, we hypothesized that using constituent cells of the niche as feeders for pre-pubertal porcine spermatogonial co-cultures would address the need for a robust long-term SSC culture system, and that exosomal exchange may mediate these effects. We used porcine spermatogonia due to the limited availability of human testis for research and the physiological similarities between pigs and humans [39,40,41]. We found that PMCs, PFFs, and Sertoli cells had similar beneficial effects on the rates of proliferation and apoptosis in prepubertal spermatogonia. In addition, we found that EVs are released by Sertoli cells and incorporated by spermatogonia in culture, and that spermatogonial proliferation decreased in response to the inhibition of exosomal release. Overall, these results show that PMCs and PFFs could serve as alternative feeders to the current Sertoli cell culture system used for porcine spermatogonia, and that the beneficial effect of feeder cells is partially mediated by exosomal exchanges between the populations.

## 2. Results

### 2.1. Establishment of Defined Feeder Cell and Spermatogonial Populations from Testis Tissue

**Figure 1:**
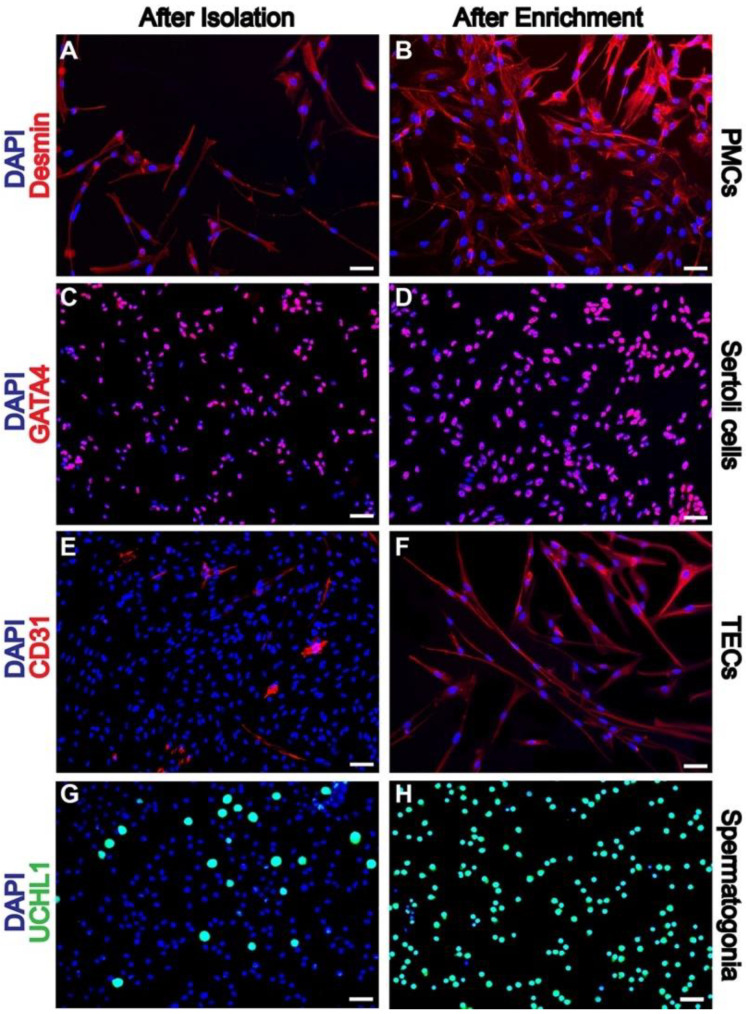
Purity of testicular feeder cells and spermatogonia increases after differential plating, passaging feeders, and FACS for spermatogonia. PMCs (**A**,**B**) (Immunofluorescence (IF) for desmin (red), DAPI (blue)), Sertoli cells (**C**,**D**) (IF for GATA4 (red), DAPI (blue)), TECs (**E**,**F**) (IF for CD31 (red), DAPI (blue)), and spermatogonia (**G**,**H**) (IF for UCHL1 (green), DAPI (blue)). DAPI was used as a counterstain for cell nuclei. Scale bars = 50 μm.

To determine which feeder cell types best supported spermatogonial culture, we first enriched different somatic cell types from one-week-old porcine testis tissue and spermatogonia from both one- and eight-week-old porcine testis tissue. Sertoli cell, PMC, TEC, and spermatogonia enrichment were optimized using different enzymatic digestions, taking advantage of differential adherence properties and passaging feeders under specific media for further enrichment. Fluorescence activated cell sorting (FACS) was used to further enrich spermatogonia [42]. Interstitial cell populations were enriched for PMCs and assessed using the PMC marker desmin [43,44], with the purity of desmin-positive cells increasing from 68.7 ± 4.6% from the initial isolation to 82.8% ± 3.6% by passage 4 (Figure 1A,B, *p* < 0.01, n = 6). Sertoli cells positive for GATA binding protein 4 (GATA4) [45,46] were enriched from starting cell populations from an initial purity of 72.9 ± 3.2% to 86.1 ± 2.5% by passage four (Figure 1C,D, *p* < 0.001, n = 7). TECs, positive for CD-31 [30,47], were passaged to increase purity from 11.9 ± 5.9% to 95.8 ± 2.1% from initial isolation to passage four (Figure 1E,F, *p* < 0.001, n = 6). The spermatogonia marker ubiquitin C- terminal hydrolase-1 (UCH-L1) [48,49,50] was used to assess the purity between initial isolation and FACS with purity increasing from 7.3 ± 3.2% to 97.6 ± 2.4% after FACS (Figure 1G,H, *p* < 0.001, n = 10).

### 2.2. Co-Culture with PMC, PFF, and Sertoli Cell Feeder Cells Supports the In Vitro Maintenance of Spermatogonia

**Figure 2:**
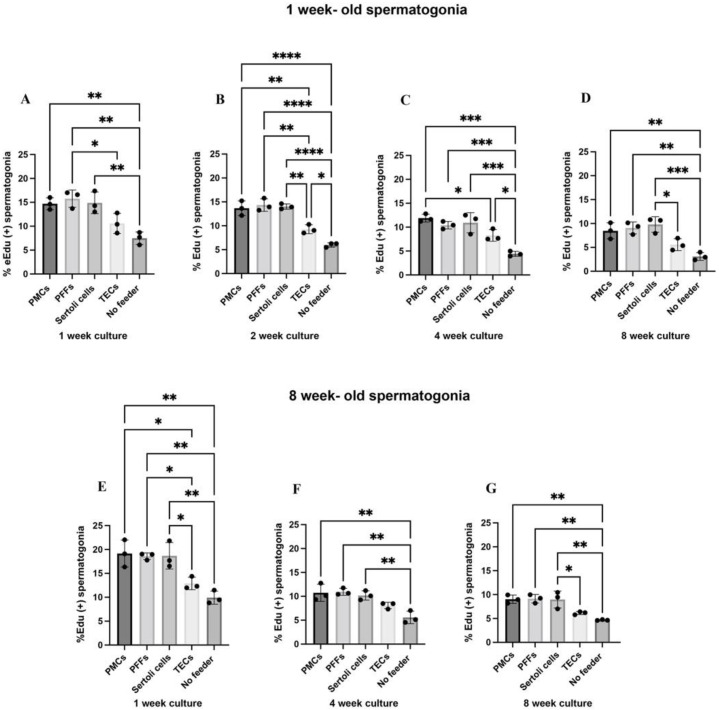
PMCs, PFFs, and Sertoli cells support higher rates of spermatogonial proliferation compared to TECs or no-feeder controls. Percent of UCH-L1 (+) spermatogonia from one-week-old porcine testis that are also EdU (+), in co-culture conditions at one week (**A**), two weeks, (**B**), four weeks (**C**), and eight weeks (**D**) of culture. Percent of UCH-L1 (+) spermatogonia from eight-week-old porcine testis that are also EdU (+), in co-culture conditions at one week (**E**), four weeks (**F**), and eight weeks (**G**) of culture. Data are presented as mean ± SD (n = 3). A *p*-value of <0.05 was considered significant (* *p* ≤ 0.05, ** *p* ≤ 0.01, *** *p* ≤ 0.001, **** *p* ≤ 0.0001).

Next, to examine the efficiency of the enriched testis-derived feeder cells, we co-cultured Sertoli cells, PMCs and TECs with spermatogonia derived from one-week and eight-week-old testis tissue. As previous studies have found murine fibroblasts to be supportive of spermatogonial proliferation in vitro [12], we also assessed porcine fibroblasts (PFFs) obtained from 40-day old porcine fetuses, as a feeder cell for co-culture. At one, two, four and eight weeks of co-culture, the proportion of proliferating spermatogonia was assessed via 5-ethynyl-2′deozyuridine (EdU) incorporation. Baseline proliferation was higher in eight-week-old spermatogonia as compared to one-week-old spermatogonia (Figure 2). Both one-week and eight-week-old spermatogonia proliferated at the highest rates on PMCs, PFFs and Sertoli cells at all time points (one-week-old: 14.70–15.77% at one week, 13.67–14.34% at two weeks, 10.41–11.88% at four weeks, and 8.48–9.80% at eight weeks; eight-week-old: 18.60–19.16% at one week, 10.19–10.94% at four weeks, and 8.97–9.13% at eight weeks, Figure 2). Proliferation of one-week-old spermatogonia on TECs was higher than in no-feeder controls at two and four-weeks of culture but was not comparable to other feeders at those timepoints (9.27 ± 0.93% vs. 5.99 ± 0.46% at two weeks, *p* < 0.05, 8.28 ± 1.22% vs. 4.42 ± 0.53% at four weeks, *p* < 0.05, Figure 2). Proliferation was lowest for spermatogonia in no-feeder controls (one-week-old: 7.49 ± 1.31% at one week, 5.99 ± 0.46% at two weeks, 4.42 ± 0.53% at four weeks, and 3.10 ± 0.81% at eight weeks, eight-week-old: 9.93 ± 1.38% at one week, 5.58 ± 1.30% at four weeks, and 4.67 ± 0.11% at eight weeks, Figure 2 indicates the ability of feeder cells to maintain spermatogonial culture in vitro. However, proliferation was lower after the four and eight-week timepoints across all feeder types (one-week-old: 8.28–11.88% at four weeks and 5.58–9.80% at eight weeks, eight-week-old: 8.09–10.94% at four weeks and 6.14–9.13% at eight weeks, Figure 2).

### 2.3. Co-Cultures with PMC, PFF, and Sertoli Cell Feeder Cells Reduce Rates of Apoptosis in Spermatogonia

**Figure 3:**
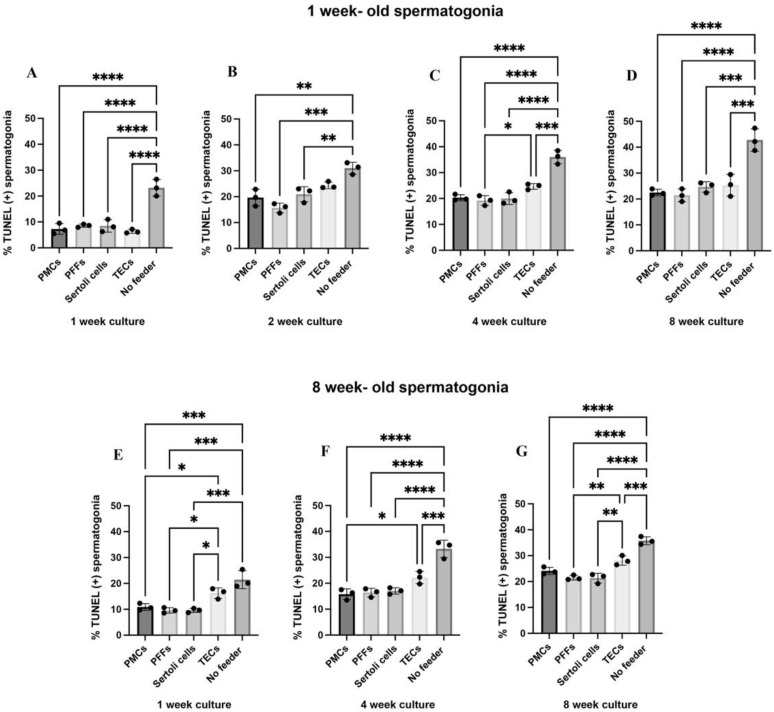
Co-culture with PMCs, PFFs, and Sertoli cells reduced apoptosis of spermatogonia. Percent of UCH-L1 (+) spermatogonia from one-week-old pigs that are also TUNEL (+), in co-culture conditions at one week (**A**), two weeks, (**B**), four weeks (**C**), and eight weeks (**D**) of culture. Percent of UCH-L1 (+) spermatogonia from eight-week-old pigs that are also TUNEL (+), in co-culture conditions at one week (**E**), four weeks (**F**), and eight weeks (**G**) of culture. Data are presented as mean ± SD (n = 3). A *p*-value of < 0.05 was considered significant (* *p* ≤ 0.05, ** *p* ≤ 0.01, *** *p* ≤ 0.001, **** *p* ≤ 0.0001).

To further investigate the role of feeders in spermatogonia co-culture we investigated the rate of apoptosis. At one, two, four and eight weeks of co-culture, the proportion of apoptotic spermatogonia was assessed using the apoptosis assay terminal deoxynucleotidyl transferase (TdT) dUTP nick-end labelling (TUNEL) and staining for UCH-L1. The rate of apoptosis was highest in one-week and eight-week-old spermatogonia across the one, two, four, and eight week time points in the no-feeder control (Figure 3). Both one-week and eight-week-old spermatogonia had the lowest rates of apoptosis on the PMCs, PFFs and Sertoli cells (Figure 3) across all time points. In the eight-week-old spermatogonia, spermatogonia co-cultured with TECs had significantly higher apoptosis than spermatogonia co-cultured with the other feeder types at one week of culture (21.46 ± 3.46% on TECs compared to 10.88 ± 1.32% on PMCs (*p* = 0.0005), 9.56 ± 1.04% on PFFs (*p* = 0.0002), and 9.58 ± 0.82% on Sertoli cells (*p* = 0.0002), n = 3, Figure 3).

### 2.4. Sertoli Cell Feeders Express the Highest Levels of GDNF, BMP4, and NELL2

**Figure 4:**
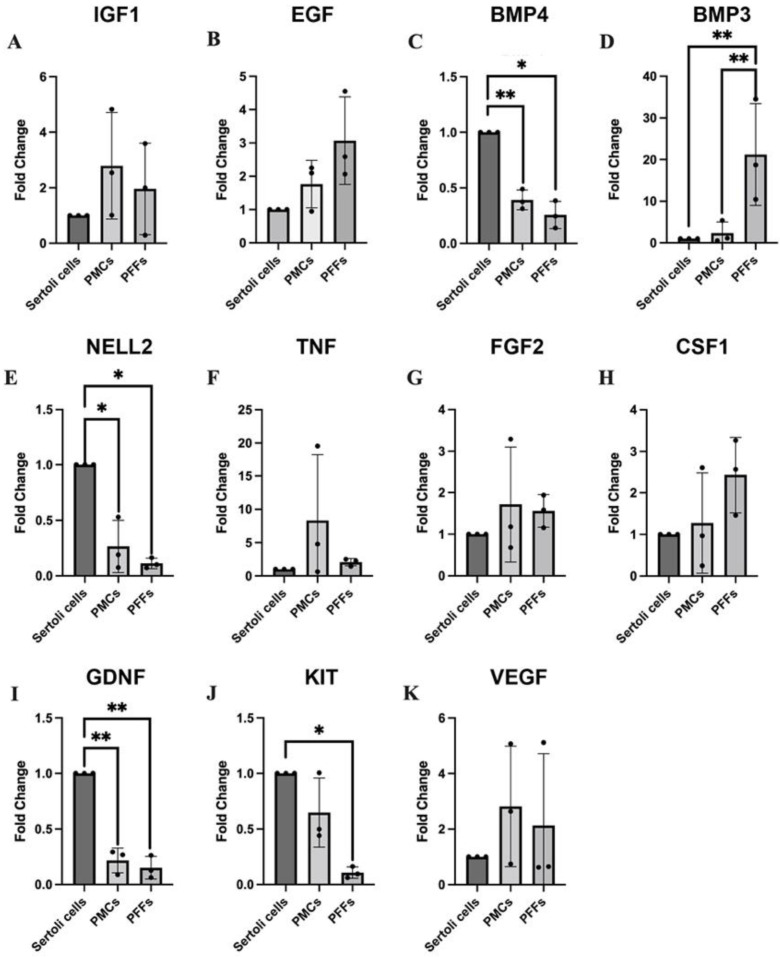
Expression of growth factors in feeders that provide similar levels of support to co-cultured spermatogonia. Levels of expression are shown as mean fold change compared to Sertoli cells (**A**) IGF1, (**B**) EGF, (**C**) BMP4, (**D**) BMP3, (**E**) NELL2, (**F**) TNF, (**G**) FGF2, (**H**) CSF1, (**I**) GDNF, (**J**) KIT, and (**K**) VEGF by Sertoli cells, PMCs, and PFFs. Data are presented as mean ± SD (n = 3). A *p*-value of < 0.05 was considered significant (* *p* ≤ 0.05, ** *p* ≤ 0.01).

PMCs, PFFs, and Sertoli cell feeders were selected as candidate feeders for further investigation as they enabled spermatogonial maintenance in vitro. To further assess the feeder cell contribution to spermatogonial maintenance in culture, growth factors known to be secreted by the feeder cells [2,22,28,51,52,53,54,55,56,57,58,59] were analysed by qRT-PCR (Figure 4). Sertoli cells expressed significantly higher levels of GDNF than PMCs and PFFs (*p* = 0.0092 and *p* = 0.0058, respectively, n = 3, Figure 4). GDNF has been identified as one of the most important factors for SSC proliferation and stem cell maintenance [20,31,54,60,61]. Sertoli cells also expressed significantly higher levels of neural EGFL like 2 (NELL2) (*p* = 0.0345 when compared with PMCs and *p* = 0.0149 when compared with PFFs, n = 3, Figure 4), as well as bone morphogenetic protein 4 (BMP4) (*p* = 0.0081 when compared with PMCs and *p* = 0.0231 when compared with PFFs, n = 3, Figure 4), which has been implicated in SSC differentiation [59]. PFFs expressed higher levels of bone morphogenetic protein 3 (BMP3) than Sertoli cells (Figure 4). BMP3 has been associated with the repression of differentiation in bone marrow stromal cells [52], and BMP signalling has been shown to be important for maintaining germline stem cells [62]. All three investigated feeder cell types expressed similar levels of epidermal growth factor (EGF), vascular endothelial growth factor (VEGF), insulin-like growth factor (IGF1), basic fibroblastic growth factor (FGF2), and colony stimulating factor 1 (CSF1).

### 2.5. Contact Co-Cultures Are Beneficial for the Proliferation of Porcine Spermatogonia

**Figure 5:**
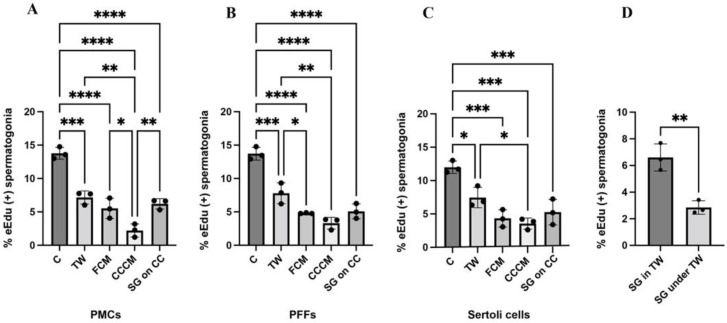
The proliferation of spermatogonia is higher in the presence of feeders than in feeder-free conditions, using conditioned media. Proliferation rates of spermatogonia grown in contact (C), in transwells (TW), using feeder-conditioned media (FCM), using co-culture conditioned media (CCCM), and grown in transwells on top of co-cultures (SG on CC) using (**A**) PMCs, (**B**) PFFs, and (**C**) Sertoli cells. (**D**) Proliferation of spermatogonia cultured feeder-free in transwells (SG in TW) and under transwells (SG under TW). Data are presented as mean ± SD (n = 3). A *p*-value of < 0.05 was considered significant (* *p* ≤ 0.05, ** *p* ≤ 0.01, *** *p* ≤ 0.001, **** *p* ≤ 0.0001).

We next investigated whether direct contact between feeder cells and spermatogonia was required for spermatogonial maintenance. Preliminary experiments (Supplementary Figure S1) showed that spermatogonia proliferated at higher rates when cultured in direct contact, compared with when they were co-cultured with physical separation using transwell inserts (13.47–15.2% in contact vs. 8.50–10.57% in transwell inserts, *p* < 0.01, Supplementary Figure S1). To better understand the underlying mechanisms, spermatogonia were cultured in different conditions to investigate how contact and separation between spermatogonia and feeders affected the proliferation of spermatogonia. Consistent with preliminary experiments, spermatogonia proliferated at the highest rates on all feeders when co-cultured in contact (13.78 ± 0.89% on PMCs, 13.72 ± 0.96% on PFFs, and 12.0 ± 0.92% on Sertoli cells, Figure 5A–C). Spermatogonia that were separated from feeders using transwell inserts also proliferated at higher rates than spermatogonia that were cultured feeder-free using co-culture-conditioned media (7.15 ± 0.99% on PMCs, 7.79 ± 1.57% on PFFs, and 7.47 ± 1.53% on Sertoli cells, Figure 5A–C). Additionally, proliferation of spermatogonia grown on a co-culture using a PMC feeder was also higher than spermatogonia grown in the PMC co-culture conditioned media (6.20 ± 0.78% on a co-culture vs. 2.21 ± 0.99% in co-culture- conditioned media, *p* = 0.0067, Figure 5A). To assess whether the use of the transwell inserts had a detrimental effect on proliferation of spermatogonia, conditions in which spermatogonia were grown feeder free in the transwell inserts or under the transwell inserts were also assessed. Interestingly, spermatogonia grown in the inserts proliferated at higher rates than those grown in the bottom of the wells with the inserts on top (6.61 ± 1.02% in the inserts vs. 2.85 ± 0.50% in the bottom of the wells with inserts on top, *p* = 0.0046, Figure 5D).

### 2.6. Exosomes Released by Sertoli Cells Are Taken up by Spermatogonia In Vitro

**Figure 6:**
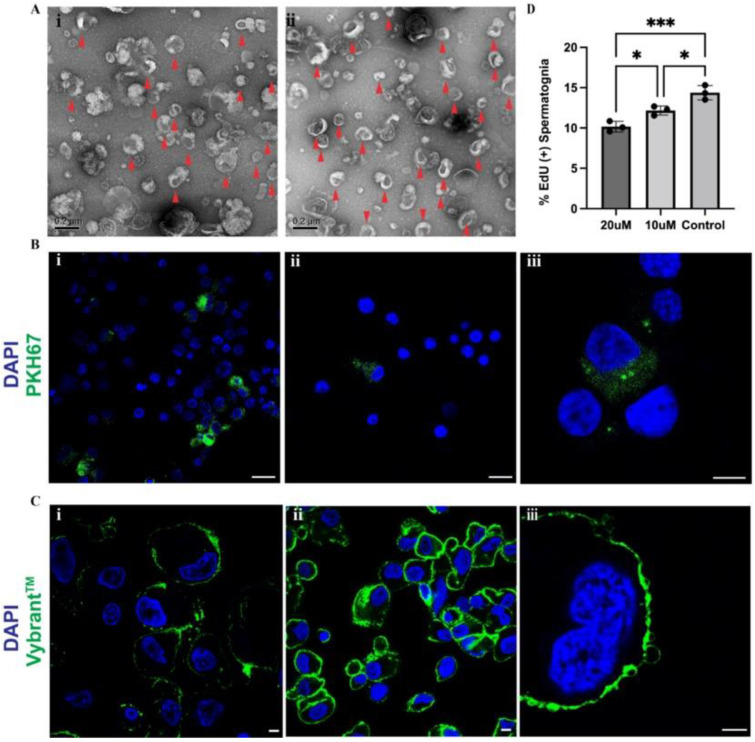
Sertoli cell-released exosomes increase spermatogonial proliferation: (**A**) Identification of Sertoli cell-isolated exosomes (red arrows) by size using TEM at 25k magnification. (**B**) Co-culture of PKH67-tagged extracellular vesicles and spermatogonia. Tagged extracellular vesicles are taken up by spermatogonia in contact (**i**,**iii**), and separated from vesicles with transwells (**ii**). (**C**) Visualization of lipid rafts on Sertoli cells that had been cultured alone for seven days (**i**), and from Sertoli cells that had been co-cultured with spermatogonia for seven days (**ii**,**iii**). (**D**) Proliferation rates of spermatogonia grown on Sertoli cell feeders treated with 10 µM of GW4869, 20 µM of GW4869, or an equivalent percentage of DMSO (control). Data are presented as mean ± SD (n = 3). A *p*-value of < 0.05 was considered significant (* *p* ≤ 0.05, *** *p* ≤ 0.001). TEM scale bars = 0.2 µm (**A**), Confocal scale bars = 25 µm (**B**) (**i**,**ii**), and 8 µm (**B**) (**iii**), and 10 µm (**C**).

Sertoli cell feeders were selected to investigate the effects of feeder-released exosomes on spermatogonia. Isolated extracellular vesicles released by Sertoli cells were characterized based on size using transmission electron microscopy (TEM) with isolated vesicles ranging in size of 30–150 nm for exosomes and 100 nm–1 µm for microvesicles (Figure 6A(i,ii)). To assess whether spermatogonia in culture incorporated Sertoli cell-released exosomes, isolated extracellular vesicles were labelled with green, fluorescent linker PKH67 and added to spermatogonia for 24 h. Exosomes were visualised in the cytoplasm of spermatogonia cultured in contact (Figure 6B(i,iii)) and in the cytoplasm of spermatogonia cultured in transwells (Figure 6B(ii)). These results indicate that spermatogonia may internalize exosomes released by Sertoli cells in vitro which may mediate Sertoli-spermatogonia communication and the beneficial effect of the feeder cells.

### 2.7. Sertoli Cell Exosome Biogenesis Involves Lipid Raft Formation

To investigate Sertoli cell exosome biogenesis, lipid raft staining was performed on Sertoli cells which were cultured in the presence of spermatogonia for one week compared to Sertoli cells cultured in isolation for the same period. The presence of lipid rafts has been implicated in exosome biogenesis and cargo sorting [63,64], and their presence is suggested to be associated with one pathway of exosome release in Sertoli cells [65,66]. Lipid rafts were observed in both Sertoli cells that had been cultured alone for seven days (Figure 6C(i)), and in Sertoli cells that had been co-cultured with spermatogonia for seven days (Figure 6C(ii,iii)). There appeared to be more staining for lipid raft formation in Sertoli cells which had been co-cultured with spermatogonia, suggesting that the co-culture may induce lipid raft formation, which may then facilitate increased Sertoli-cell exosomal release.

### 2.8. Inhibition of Sertoli Cell Exosome Release Reduces Spermatogonial Proliferation In Vitro

To investigate how Sertoli cell-released exosomes affect spermatogonia, the exosomal inhibitor GW4869 was used to inhibit Sertoli cell exosomal release. GW4869 is a small molecule that inhibits exosome generation through the inhibition of sphingomyelinase [67,68,69] which is involved in lipid raft-mediated exosome biogenesis. After seven days, the proliferation of spermatogonia co-cultured with Sertoli cells with GW4869 was significantly lower than in the control (Figure 6D). Proliferation of spermatogonia co-cultured with 20 µM of GW4869 was lowest (10.17 ± 0.65%), with spermatogonia co-cultured with 10 µM of GW4869 proliferating at a significantly higher rate (12.17 ± 0.57%, *p* = 0.03), and spermatogonia co-cultured with DMSO proliferating at the highest rate (14.38 ± 0.90%, *p* = 0.0009 and *p* = 0.022 for control vs. 20 µM and control vs. 10 µM, respectively) (Figure 6D), indicating that exosomal release contributes to the survival of spermatogonia.

## 3. Discussion

The establishment of long term spermatogonial culture for larger animals has remained challenging, unable to replicate the success achieved in rodents [12,13,15,16]. The development of a culture system supporting the maintenance and proliferation of spermatogonia in vitro from translational models, such as pigs [39,40,41], is a critical step towards human SSC expansion, a prerequisite to the development of therapies for fertility preservation for prepubertal male cancer patients. However, the precise factors required to support porcine spermatogonia have not yet been conclusively identified. Collectively, our data suggest that spermatogonial maintenance is best achieved using co-cultures with feeder cells resident in the SSC niche, Sertoli cells and PMCs, and fetal fibroblasts (PFFs). We found that porcine Sertoli cells express higher levels of GDNF than PMCs and PFFs, which are critical for SSC self-renewal, and other factors such as BMP4 and NELL2. Additionally, contact co-cultures sustain higher spermatogonial proliferation than indirect feeder co-cultures. Furthermore, we identified that exosomes released by Sertoli cells are taken up by spermatogonia in vitro, and that inhibition of Sertoli cell exosomal release reduced spermatogonial proliferation.

Previous research by Zhang et al., 2017 found that porcine spermatogonia proliferated for over eight weeks, expressing markers for stem cells and undifferentiated cells on neonatal Sertoli cell feeder layers [25]. Sertoli cells have also been identified as the optimum feeder cells for goat spermatogonia culture [70]. However, other cells from the SSC niche shown to support SSCs in vitro and in vivo [30,31,47,71] have not been investigated. In vivo, PMCs and Sertoli cells form the cellular boundary of the SSC niche [71]. PMCs may contribute to SSC maintenance via GDNF secretion, with peritubular cell-derived GDNF found to be crucial for lifelong spermatogenesis in mice [31,71]. TECs also secrete GDNF, and transplantation of TECs into SSC depleted mouse testes restored spermatogenesis [30]. Additionally, murine and bovine fibroblastic cells have been used to culture rodent and bovine spermatogonia, respectively [12,15,72], warranting further investigation of species-specific fibroblastic feeder cells. Our data shows the highest rates of proliferation and the lowest rates of apoptosis in spermatogonia co-cultured with PMCs, PFFs, and Sertoli cells. While TECs also produce GDNF [30] and other factors that support SSCs, they had the lowest ability to support spermatogonial maintenance. Previous research has suggested that SSCs may be positioned preferentially within regions proximal to interstitial tissue and vasculature [72,73]; however, research by Chan et al., 2014 suggested that the SSC pool in the testes of adult mice was distributed in avascular regions of the seminiferous tubules [74], which may be one reason why TECs isolated in our study were insufficient feeder cells.

Despite initially supporting the maintenance and proliferation of both one- and eight-week-old porcine spermatogonia at rates comparable to murine spermatogonia co-cultured with a fetal fibroblast cell line [75], both one- and eight-week spermatogonia co-cultured on PMCs, PFFs and Sertoli cells had reduced proliferative capacity and increased apoptosis after four weeks of culture, with proliferation further reduced by termination of culture at eight weeks. Each feeder has a distinct secretome that may not fully recapitulate the synergistic secretions of the SSC niche. Here, feeder populations were derived from one-week-old prepubertal testis and were enriched for specific cell types to generate a homogenous feeder quite different from the heterogenous SSC niche in vivo [27]. Thus, in further optimising co-culture, multi-layer heterogenous feeder cells should be investigated to determine whether this would facilitate longer term culture. Nonetheless, this study is one step forward toward improving spermatogonia culture conditions and understanding mechanisms that support their proliferation in culture.

In the current study, while both one- and eight-week-old porcine spermatogonia had similar feeder responses, the absolute values for proliferation at one week of culture were higher in eight-week-old spermatogonia. Spermatogonia from one- and eight-week-old pigs have different metabolic requirements [76], with one-week-old spermatogonia preferentially consuming pyruvate for subsequent OXPHOS and transition to glycolysis by eight-weeks, and therefore they may respond differently to identical culture conditions. These findings are of particular interest, as feeder cells were derived from one-week old testis tissue or fetal fibroblasts, and as such could be expected to represent in vivo conditions more closely for the one-week-old spermatogonial cultures than for the eight-week-old spermatogonial cultures. Feeder cells were derived from one-week-old testis tissues, as previous research has shown that neonatal Sertoli cells were better able to support prepubertal porcine spermatogonia proliferation and maintenance than adult Sertoli cells [25], indicating that younger feeders may be more supportive in general than older feeders. Nevertheless, the reduced proliferation of one-week old spermatogonia in age-matched feeders suggest that they may require additional niche-derived factors that are not required by eight-week-old spermatogonia, or that eight-week-old spermatogonia are inherently more proliferative at a basal level. Comparisons of spermatogonial proliferation in stirred suspension bioreactors (SSBs) and in static cultures indicate that it may be the latter, as eight-week-old spermatogonia similarly had higher levels of proliferation than one-week-old spermatogonia in the feeder- free static cultures and in the bioreactors [75]. By four weeks of culture, however, proliferation was comparable between both ages and the rates of apoptosis were similar across all timepoints between both ages of spermatogonia.

To work toward future feeder free culture, we investigated whether the ability of feeders to support spermatogonia in vitro is through contact. Here we found that across feeder types, spermatogonia proliferated at higher rates in contact with feeders as compared with separation via a transwell insert. Feeder cells provide structural support to spermatogonia, which may mimic the basement membrane which is generally required for maintenance of stemness both in vitro and in vivo [77,78,79]. This regulation of stem cell fate is mediated by mechanical, adhesive, and topological cues interacting with dynamic soluble factors [80,81,82] where the biophysical cues can initiate mechanotransduction inducing intracellular biochemical and functional responses by stem cells [83,84]. Feeder-free systems have recapitulated this structural support by using hydrogels including Matrigel [19], agarose [85], poly-L-lysine and laminin [86,87]. Spermatogonia cultured in transwells were not provided with similar structural support, which may have in turn modulated their response to soluble factors, reducing their proliferation.

Notably, however, spermatogonia in transwells proliferated at higher rates than spermatogonia in conditioned media in the absence of feeders. This suggests feeder-specific exchange which is not recapitulated by conditioned media alone. This may be explained by differential exosome cargo sorting in feeders in co-culture conditions compared with feeders cultured alone, as culture conditions can affect cargo sorting. For example, hepatocyte culture supernatants were found to be enriched for heat shock protein 70 (Hsp 70) after receiving a 1 h heat shock [88]. Similarly, exposure to stressors including hypoxia, inflammation, and high glucose had an effect on the mRNA profiles of exosomes released from human microvascular endothelial cells [89]. The quantity of exosomes released from feeders may also change in the presence of spermatogonia. Inhibition of mTORc1 via treatment with rapamycin has been shown to promote exosomal release from MEFs [90]. While the addition of spermatogonia to feeders may not cause significant cellular stress, it is possible that their presence affects the quantity and contents of feeder-derived exosomes through intercellular communication that can only occur in co-culture conditions, which is likely facilitated through contact. Further investigations characterizing feeder released exosomal cargo, both in contact and separated, are required to wholly elucidate this process.

The role of exosomes within the testes is largely unknown [91], however a recent study by Ma et al., 2022 found that Sertoli cells released exosomes in vivo, delivering their contents to Leydig cells modulating Leydig cell survival through CCL20. Additionally, it has been reported that Sertoli cell-released exosomes mediate the transfer of miR-486-5p to SSCs and promote differentiation by the upregulation of Stra8 in vitro [92]. Due to the beneficial nature of Sertoli cell feeders on spermatogonial maintenance in vitro as observed in this study and others [25,70], we hypothesised that Sertoli-spermatogonia communication may be at play. We found that Sertoli cell-released exosomes were taken up by spermatogonia in culture, and that spermatogonia were observed to take up less tagged exosomes when they were separated from the tagged isolates using transwell inserts, indicating that exosomal exchange is more efficient in cells which are in proximity. As previously hypothesized, this is likely contributing to the lower rates of proliferation of spermatogonia co-cultured in transwell inserts. Furthermore, we observed lipid rafts present on Sertoli cells which have been indicated as a pathway of exosomal biogenesis [63]. Lipid rafts were found in Sertoli cells that had been cultured alone for seven days, and in Sertoli cells that had been co-cultured with spermatogonia for seven days. However, as more lipid rafts appeared to be present in the co-cultured Sertoli cells, suggesting that the presence of spermatogonia may induce lipid raft formation in Sertoli cells potentially through feedback in the form of exosomes released from spermatogonia. The application of the small molecule exosomal inhibitor GW4869 to Sertoli cell spermatogonia co-culture, a sphingomyelinase inhibitor which is involved in an exosome biogenesis pathway [67,93], reduced spermatogonial proliferation. These results indicate that Sertoli cell-spermatogonia communication via the transfer of extracellular vesicles may be essential for spermatogonial maintenance in vitro and may be partly responsible for the failure of feeder-free culture conditions to support larger animal spermatogonial culture. However, additional studies are required to assess spermatogonia-released exosomes and whether they in turn influence Sertoli cells.

## 4. Materials and Methods

Reagents are listed in Supplementary Information (Table S1).

### 4.1. Experimental Model and Sample Details

Testes were obtained from Sunterra Farms Ltd. (Acme, AB, Canada) and the University of Alberta, Edmonton, AB, from castration of one- and eight-week-old piglets (*sus scrofra*), respectively. All experimental procedures were carried out with the approval and oversight of the University of Calgary’s Institutional Animal Care Committee.

### 4.2. Feeder Cell and Spermatogonia Isolation and Enrichment

Cell populations for feeder cells, Sertoli cells, PMCs and TECs, were isolated from one-week-old testes. PMCs and TECs were isolated using a modified protocol established for rat PMC isolation [43]. Briefly, harvested testes were decapsulated and shredded into 1–2 mm pieces of tubules using forceps. Tubule pieces were then washed using Hank’s Balanced Saline Solution (HBSS) (Sigma, St. Louis, MO, USA) w/1% Penicillin streptomycin (P/S) (Sigma, St. Louis, MO, USA) and digested using collagenase type IV (1 mg/mL; Worthington Biochemical Corporation, Lakewood, NJ, USA) for 15 min at 37 °C in Dulbecco’s Modified Eagle Medium (DMEM) (Sigma, St. Louis, MO, USA). During the digestion, tubes containing the tissue and enzymes were inverted every 2–5 min. The resulting cell suspension, consisting of mostly of interstitial cells, was then filtered through a 70-µm mesh and 40-µm mesh, and then centrifuged at 500× *g* for 5 min. Cells were then resuspended and seeded onto 100 mm culture plates in DMEM supplemented with 10% fetal bovine serum (FBS) (ThermoFisher, Grand Island, NY, USA), 1% P/S, and 1% L- glutamine (Sigma, St. Louis, MO, USA). Seeded cells were left in a 21% O_2_, 37 °C incubator overnight, and media was replaced in the morning to remove non-adherent cells including erythrocytes and Leydig cells. Plated cells were then removed from plates with 0.25% Trypsin- EDTA (ThermoFisher, Grand Island, NY, USA), and a biotinylated antibody against CD-31 (Abcam, Rockford, IL, USA) was added and incubated with cells at room temperature (RT) for 15 min. The TEC marker CD-31 [30,47] was used to separate TECs from PMCs and other contaminating populations using magnetic activated cell sorting (MACS) and the EasySep™ Release Biotin Positive Selection Kit (STEM CELL Technologies, Vancouver, BC, Canada). The cells were passaged in cell-type- specific media after isolation to increase purity. The CD-31 positive cell-population, consisting of TECs and contaminating populations, were grown in endothelial cell growth media with growth medium supplement mix and 1% P/S. The flow-through cells, consisting of PMCs and contaminating populations, were grown in DMEM with 20% FBS and 1% P/S. Sertoli cells and spermatogonia were isolated from the testis by a two-step enzymatic digestion, as previously described [42]. Testes were decapsulated and cut into 1–2 mm pieces using scissors. The tissue was then digested twice, for 30 min each, in collagenase type IV. Digestion tubes were inverted every 5–7 min. The tissue was then washed in DPBS and tubules were collected into 50 mL tubes and digested in 0.25% Trypsin- EDTA until mostly single cells were visible when looking at the digested tissue under a microscope. The initial testicular cell population was differentially plated to enrich for Sertoli cells and spermatogonia through adherence properties of the individual cell type. A final cell population containing spermatogonia was enriched using FACS based on forward and side scatter properties as previously described [94].

### 4.3. Immunofluorescence

The purity of isolated PMCs, TECs, Sertoli cells, and spermatogonia was assessed after initial isolation and after sorting or passage four, using antibodies against cell markers UCH-L1 (1:200; Abcam, Waltham, MA, USA), GATA-4 (1:200; Santa Cruz Biotechnology, Dallas, TX, USA), desmin (1:200; Abcam, Waltham, MA, USA) and CD-31 (1:100; Abcam, Waltham, MA, USA), respectively. Cells were fixed with 2% paraformaldehyde (PFA) at RT for 15 min and permeabilized in 0.2% Triton-X (Sigma, St. Louis, MO, USA) in PBS for 10 min at RT. Cells were then blocked with CAS-block (ThermoFisher, Grand Island, NY, USA) and incubated with the primary antibody at 4 °C overnight. The samples were then incubated with secondary antibodies conjugated with Alexa Fluor 594 for feeders and Alexa Fluor 488 for spermatogonia (1:500) for 1 h at RT. The nuclei were stained with DAPI (4′,6-diamidino-2-phenylindole) and samples were imaged with a fluorescence microscope, and the number and percentage of cells were quantified using Image J by counting approximately 400 cells per sample.

### 4.4. Co-Culture Conditions

Enriched feeder cells, PMCs, TECs, Sertoli cells, and PFFs (RCI, Egan, MN, USA), were treated with mitomycin-c (Sigma, St. Louis, MO, USA) in DMEM with 1% P/S and incubated at 37 °C for 3 h before collection with 0.25% Trypsin- EDTA. The feeder cells were then seeded onto 6-well plates, pre-coated with 0.1% gelatin (Sigma, St. Louis, MO, USA, at a seeding density of 6.5 × 10^4^ cells/mL (6.5 × 10^4^ cells in 1 mL of media in each well) and cultured in α-MEM with additives [76] at 37 °C for 18 h. Spermatogonia were seeded onto feeder cells at a seeding density of 5.0 × 10^5^ cells/mL. The spermatogonia co-cultures were cultured for a total of one, two, four, or eight weeks, with media changes every 48 h and spermatogonia passaged to new feeders every 7 d using 1:20 trypsin. For the last 12 h of culture, spermatogonia were exposed to EdU (5-ethynyl-2′deozyuridine) to quantify the proportion of cells actively synthesizing DNA during that period. Cells were then harvested using 0.25% trypsin EDTA.

### 4.5. EdU Incorporation Assay and TUNEL Staining

EdU uptake was visualized according to manufacturer’s instructions using the Click-iT™ EdU Alexa Fluor 488 imaging kit (ThermoFisher, Grand Island, NY, USA). Spermatogonia were then co- stained for UCH-L1, a marker for undifferentiated spermatogonia [95]. The proportion of UCH-L1 positive cells that were also EdU positive were imaged using fluorescence microscopy and quantified by counting approximately 400 cells per sample using ImageJ.

Samples for TUNEL staining were fixed in 2% PFA and stained using the Click-iT™ TUNEL Alexa Fluor™ 488 Imaging Assay kit (ThermoFisher, Grand Island, NY, USA) according to the manufacturer’s instructions. Cells were then co-stained for UCH-L1 and imaged using fluorescence microscopy. The proportion of UCH-L1 positive cells that were also TUNEL- positive was quantified by counting approximately 400 cells per sample using ImageJ.

### 4.6. qRT-PCR

Gene expression of growth factors associated with promoting SSC proliferation, stem cell maintenance, and differentiation was assessed via qRT-PCR (Table S4), as previously described [76]. RNA was extracted from 1.0 × 10^6^ cells using the RNeasy Mini Kit (Qiagen, Toronto, ON, Canada). 2 μg of total RNA was used for reverse transcription, using MultiScribe Reverse Transcriptase (Applied Biosystems, ThermoFisher Scientific, Burlingtn, ON, Canada). cDNA concentrations were measured for each sample and was diluted 25-fold to 2.55 ng for q-PCR using 7.500 Fast Real Time PCR System (Applied Biosystems, ThermoFisher Scientific, Burlington, ON, Canada), SsoFast Eva Green Supermix with Low ROX (Bio-Rad, Mississauga, ON, Canada). Gene expression was analyzed after results were normalized to the mean of the internal controls (HPRT1, RPL4, EIFl3).

### 4.7. Transwell Spermatogonia Co-Cultures

Spermatogonia from one-week-old porcine testis were cultured in 24-well plates, using a seeding density of 1.3 × 10^4^ feeder cells (PMC, PFF or Sertoli cell feeders) and 1.0 × 10^5^ spermatogonia per well. For each condition (Table S3), four to six wells were prepared with α-MEM as previously described [76] and were cultured at 37 °C and 21% O_2_. Experimental conditions using feeder- and co-culture- conditioned media required half of the media to be changed every 24 h so cultured spermatogonia would not be receiving media that was depleted of additives to the basal media. Half of the media was changed every 24 h for other conditions as well, to maintain consistency between conditions. After one week, for the last 12 h of culture spermatogonia were exposed to EdU (5-ethynyl-2′deozyuridine) to quantify the proportion of cells actively synthesizing DNA during that period. Cells were then harvested using 0.25% trypsin EDTA.

### 4.8. Extracellular Vesicle Isolation

Extracellular vesicles were isolated from Sertoli cells from the first differential plating via ultracentrifugation, as previously described [96]. Briefly, following removal of media and unadhered cells, exosomal collection media; α-MEM (ThermoFisher, Grand Island, NY, USA) with 1%P/S, 1% Na Pyruvate (ThermoFisher, Grand Island, NY, USA) 1% L-Glutamine, and 1.5% HEPES buffer (Sigma, St. Louis, MO, USA), was added to the adhered Sertoli cells for culture at 37 °C at 21% O_2_ for 72 h. The media was then collected and centrifuged at increasing forces of 300× *g* for 10 min, 2000× *g* for 10 min, 10,000× *g* for 30 min and 100,000× *g* for 70 min. Before the final centrifugation, the isolate was filtered through a 0.2 µm syringe filter, and the filtered isolate was ultracentrifuged again for 70 min at 100,000× *g*. The resulting pellet was resuspended in 50–150 µL of PBS.

### 4.9. TEM of Extracellular Vesicles

Extracellular vesicle isolates were resuspended in 150 µL of PBS and fixed in 450 µL of 2.5% glutaraldehyde. 5 µL of fixed isolates were then placed on glow-discharged formvar-carbon coated grids. Grids were washed three to four times in 100 mM, and then 10 mM ammonium acetate, and then stained with 50 µL of 1% uranyl acetate. Grids were left to dry at RT and were immediately visualized using the Tecnai F20 (Thermo Fisher Scientific, Waltham, MA, USA).

### 4.10. Extracellular Vesicle Labelling

Isolated exosomes were labeled for tracing studies. Exosomes released from Sertoli cells were collected and labelled with green fluorescent PKH67 as per manufacturer’s instructions. The isolated exosomes were diluted in 1 mL of diluent C and incubated with 2 μL of PKH67 staining solution (Sigma, St. Louis, MO, USA) for 5 min at RT. Unbound dye was quenched by 5 mL of 1% BSA (Sigma, St. Louis, MO, USA) and exosomes were harvested again via ultracentrifugation at 100,000× *g* for 2 h at 4 °C. PKH67-labeled exosomes were added to 1.0 × 10^5^ spermatogonia in the wells of a 24-well plate, both with and without transwell inserts, for 24 h at 37 °C (90 µL/mL). Cells were washed twice with PBS, fixed with 2% PFA for 15 min at RT and stained with 4′,6-diamidino-2-phenylin-dole (DAPI).

### 4.11. Treatment of Sertoli Cells with GW4869

6.5 × 10^4^ Sertoli cells/mL 6.5 × 10^4^ per well were seeded into the wells of a 6- well plate with α-MEM (Table S1) containing either 10 µM or 20 µM of GW486 (Sigma, St. Louis, MO, USA). Sertoli cells plated in media containing only DMSO were used as a negative control. After one week in culture, EdU was added to spermatogonia for the last 12 h of culture before collection with 0.25% Trypsin- EDTA, double-staining for UCH-L1 and imaging and quantification, as previously described.

### 4.12. Lipid Raft Labelling

Sertoli cells that had been either cultured alone, or co-cultured with one-week-old spermatogonia for one week were collected from 6-well plates with 0.25% Trypsin- EDTA, and the Vybrant™ Alexa Fluor™ 488 Lipid Raft Labeling Kit (ThermoFisher, Grand Island, NY, USA) was used, as per the manufacturer’s instructions, to label lipid rafts in the samples. Samples were then fixed with 2% PFA in PBS, and counter stained with DAPI. Samples were visualized using a Leica TCS SP5 confocal microscope (Leica microsystems, Wetzlar, Germany).

### 4.13. Statistical Analysis

Data is presented as mean ± SD, if not otherwise stated, from a minimum of three biological replicates. Statistics for proliferation and apoptosis rates were calculated using Brown-Forsythe and Welch one-way ANOVA tests with Tukey’s multiple comparisons test. Statistics for gene expression ∆ CT values were calculated using unpaired parametric t-tests with Welch’s correction unless data was found not to be normally distributed through Shapiro- Wilk tests; in which case Mann-Whitney tests were used. A value of *p* < 0.05 was considered statistically significant. Statistical analyses were performed using GraphPad Prism 9.3.1 Software (San Diego, CA, USA).

## 5. Conclusions

Our studies provide insight into co-culture systems which enable spermatogonia maintenance, and furthermore shed light on how exosomal exchange may support spermatogonial self-renewal. Our results demonstrate that Sertoli cells, PMCs and PFFs are suitable feeder cells and that Sertoli cell-released exosomes can promote the proliferation of spermatogonia in vitro. These findings are key in directing further characterization of the dynamic cargo released by feeder cells, which may ultimately allow the development of feeder-free culture systems for clinical use.

## Data Availability

The data that support the findings of this study are available upon request from the corresponding author.

## Supplementary Materials

The following supporting information can be downloaded at: https://www.mdpi.com/article/10.3390/ijms23094535/s1.

## References

[1] Kostereva N., Hofmann M.C. (2008). Regulation of the Spermatogonial Stem Cell Niche. Reprod. Domest. Anim..

[2] Oatley J.M., Brinster R.L. (2008). Regulation of Spermatogonial Stem Cell Self-Renewal in Mammals. Annu. Rev. Cell Dev. Biol..

[3] Oatley J.M., Brinster R.L. (2006). Spermatogonial Stem Cells. Methods Enzymol..

[4] Morrison S.J., Spradling A.C. (2008). Stem Cells and Niches: Mechanisms That Promote Stem Cell Maintenance throughout Life. Cell.

[5] Takashima S., Shinohara T. (2018). Culture and transplantation of spermatogonial stem cells. Stem Cell Res..

[6] Kubota H., Brinster R.L. (2006). Technology insight: In vitro culture of spermatogonial stem cells and their potential therapeutic uses. Nat. Clin. Pract. Endocrinol. Metab..

[7] Brinster R.L., Avarbock M.R. (1994). Germline transmission of donor haplotype following spermatogonial transplantation. Proc. Natl. Acad. Sci. USA.

[8] Brinster R.L., Zimmermann J.W. (1994). Spermatogenesis following male germ-cell transplantation. Proc. Natl. Acad. Sci. USA.

[9] Tagelenbosch R.A.J., de Rooij D.G. (1993). A quantitative study of spermatogonial multiplication and stem cell renewal in the C3H/101 F1 hybrid mouse. Mutat. Res.-Fundam. Mol. Mech. Mutagene..

[10] Hermann B.P., Phillips B.T., Orwig K.E. (2011). The Elusive Spermatogonial Stem Cell Marker?. Biol. Reprod..

[11] Aponte P.M. (2015). Spermatogonial stem cells: Current biotechnological advances in reproduction and regenerative medicine. World J. Stem Cells.

[12] Kanatsu-Shinohara M., Ogonuki N., Inoue K., Miki H., Ogura A., Toyokuni S., Shinohara T. (2003). Long-Term Proliferation in Culture and Germline Transmission of Mouse Male Germline Stem Cells. Biol. Reprod..

[13] Kanatsu-Shinohara M., Muneto T., Lee J., Takenaka M., Chuma S., Nakatsuji N., Horiuchi T., Shinohara T. (2008). Long-Term Culture of Male Germline Stem Cells From Hamster Testes. Biol. Reprod..

[14] Kubota H., Brinster R.L. (2008). Culture of Rodent Spermatogonial Stem Cells, Male Germline Stem Cells of the Postnatal Animal. Methods Cell Biol..

[15] Ryu B.Y., Kubota H., Avarbock M.R., Brinster R.L. (2005). Conservation of spermatogonial stem cell self-renewal signaling between mouse and rat. Proc. Natl. Acad. Sci. USA.

[16] Kubota H., Wu X., Goodyear S.M., Avarbock M.R., Brinster R.L. (2011). Glial cell line-derived neurotrophic factor and endothelial cells promote self-renewal of rabbit germ cells with spermatogonial stem cell properties. FASEB J..

[17] Kanatsu-Shinohara M., Inoue K., Ogonuki N., Morimoto H., Ogura A., Shinohara T. (2011). Serum- and Feeder-Free Culture of Mouse Germline Stem Cells. Biol. Reprod..

[18] Kanatsu-Shinohara M., Miki H., Inoue K., Ogonuki N., Toyokuni S., Ogura A., Shinohara T. (2005). Long-Term Culture of Mouse Male Germline Stem Cells Under Serum-or Feeder-Free Conditions. Biol. Reprod..

[19] Choi N.Y., Park Y.S., Ryu J.S., Lee H.J., Araúzo-Bravo M.J., Ko K., Han D.W., Schöler H.R., Ko K. (2014). A novel feeder-free culture system for expansion of mouse spermatogonial stem cells. Mol. Cells.

[20] Kanatsu-Shinohara M., Ogonuki N., Matoba S., Morimoto H., Ogura A., Shinohara T. (2014). Improved Serum- and Feeder-Free Culture of Mouse Germline Stem Cells. Biol. Reprod..

[21] Kanatsu-Shinohara M., Ogonuki N., Iwano T., Lee J., Kazuki Y., Inoue K., Miki H., Takehashi M., Toyokuni S., Shinkai Y. (2005). Genetic and epigenetic properties of mouse male germline stem cells during long-term culture. Development.

[22] Aponte P.M., Soda T., Teerds K.J., Mizrak S.C., van de Kant H.J.G., de Rooij D.G. (2008). Propagation of bovine spermatogonial stem cells in vitro. Reproduction.

[23] Kong L., Qiu L., Guo Q., Chen Y., Zhang X., Chen B., Zhang Y., Chang G. (2018). Long-term in vitro culture and preliminary establishment of chicken primordial germ cell lines. PLoS ONE.

[24] Suyatno, Kitamura Y., Ikeda S., Minami N., Yamada M., Imai H. (2018). Long-term culture of undifferentiated spermatogonia isolated from immature and adult bovine testes. Mol. Reprod. Dev..

[25] Zhang P., Chen X., Zheng Y., Zhu J., Qin Y., Lv Y., Zeng W. (2017). Long-Term Propagation of Porcine Undifferentiated Spermatogonia. Stem Cells Dev..

[26] Lee K.H., Lee W.Y., Kim J.H., Park C.K., Do J.T., Kim J.H., Choi Y.S., Kim N.H., Song H. (2016). Subculture of germ cell-derived colonies with gata4-positive feeder cells from neonatal pig testes. Stem Cells Int..

[27] Oatley J.M., Brinster R.L. (2012). The Germline Stem Cell Niche Unit in Mammalian Testes. Physiol. Rev..

[28] Maekawa M., Kamimura K., Nagano T. (1996). Peritubular myoid cells in the testis: Their structure and function. Arch. Histol. Cytol..

[29] Nakagawa T., Nabeshima Y.-I., Yoshida S. (2007). Functional Identification of the Actual and Potential Stem Cell Compartments in Mouse Spermatogenesis. Dev. Cell.

[30] Bhang D.H., Kim B.J., Kim B.G., Schadler K., Baek K.H., Kim Y.H., Hsiao W., Ding B.S., Rafii S., Weiss M.J. (2018). Testicular endothelial cells are a critical population in the germline stem cell niche. Nat. Commun..

[31] Chen L.-Y., Willis W.D., Eddy E.M. (2016). Targeting the Gdnf Gene in peritubular myoid cells disrupts undifferentiated spermatogonial cell development. Proc. Natl. Acad. Sci. USA.

[32] Spinnler K., Köhn F.M., Schwarzer U., Mayerhofer A. (2010). Glial cell line-derived neurotrophic factor is constitutively produced by human testicular peritubular cells and may contribute to the spermatogonial stem cell niche in man. Hum. Reprod..

[33] Raposo G., Stoorvogel W. (2013). Extracellular vesicles: Exosomes, microvesicles, and friends. J. Cell Biol..

[34] Catalano M., O’Driscoll L. (2019). Inhibiting extracellular vesicles formation and release: A review of EV inhibitors. J. Extracell. Vesicles.

[35] Zaborowski M.P., Balaj L., Breakefield X.O., Lai C.P. (2015). Extracellular Vesicles: Composition, Biological Relevance, and Methods of Study. Bioscience.

[36] Hessvik N.P., Llorente A. (2018). Current knowledge on exosome biogenesis and release. Cell. Mol. Life Sci..

[37] Théry C. (2011). Exosomes: Secreted vesicles and intercellular communications. F1000 Biol. Rep..

[38] Zhang Y., Liu Y., Liu H., Tang W.H. (2019). Exosomes: Biogenesis, biologic function and clinical potential. Cell Biosci..

[39] Vodička P., Smetana K., Dvořánková B., Emerick T., Xu Y.Z., Ourednik J., Ourednik V., Motlík J. (2005). The miniature pig as an animal model in biomedical research. Ann. N. Y. Acad. Sci..

[40] González R., Dobrinski I. (2015). Beyond the mouse monopoly: Studying the male germ line in domestic animal models. ILAR J..

[41] Dawson H.D., Smith A.D., Chen C., Urban J.F. (2017). An in-depth comparison of the porcine, murine and human inflammasomes; lessons from the porcine genome and transcriptome. Vet. Microbiol..

[42] Sakib S., Yu Y., Voigt A., Ungrin M., Dobrinski I. (2019). Generation of Porcine Testicular Organoids with Testis Specific Architecture using Microwell Culture. J. Vis. Exp..

[43] Tung P.S., Fritz I.B. (1990). Characterization of Rat Testicular Peritubular Myoid Cells in Culture: α-Smooth Muscle Isoactin is a Specific Differentiation Marker. Biol. Reprod..

[44] Maretta M., Marettová E. (2004). Immunohistochemical demonstration of myoid cells in the testis and its excurrent ducts in the domestic fowl. Br. Poult. Sci..

[45] Kyrönlahti A., Euler R., Bielinska M., Schoeller E.L., Moley K.H., Toppari J., Heikinheimo M., Wilson D.B. (2011). GATA4 regulates Sertoli cell function and fertility in adult male mice. Mol. Cell. Endocrinol..

[46] Ketola I., Pentikäinen V., Vaskivuo T., Ilvesmäki V., Herva R., Dunkel L., Tapanainen J.S., Toppari J., Heikinheimo M. (2000). Expression of Transcription Factor GATA-4 during Human Testicular Development and Disease. J. Clin. Endocrinol. Metab..

[47] Kim Y.-H., Oh M.-G., Bhang D.H., Kim B.-J., Jung S.-E., Kim S.-M., Dohr G., Kim S.-U., Ryeom S., Ryu B.-Y. (2019). Testicular endothelial cells promote self-renewal of spermatogonial stem cells in rats. Biol. Reprod..

[48] Luo J., Megee S., Rathi R., Dobrinski I. (2006). Protein gene product 9.5 is a spermatogonia-specific marker in the pig testis: Application to enrichment and culture of porcine spermatogonia. Mol. Reprod. Dev..

[49] He Z., Kokkinaki M., Jiang J., Dobrinski I., Dym M. (2010). Isolation, Characterization, and Culture of Human Spermatogonia1. Biol. Reprod..

[50] Alpaugh W.F., Voigt A.L., Dardari R., Su L., Al Khatib I., Shin W., Goldsmith T.M., Coyle K.M., Tang L.A., Shutt T.E. (2021). Loss of Ubiquitin Carboxy-Terminal Hydrolase L1 Impairs Long-Term Differentiation Competence and Metabolic Regulation in Murine Spermatogonial Stem Cells. Cells.

[51] Ryu B.-Y., Nagano M., Brinster C.J., Avarbock M.R., Brinster R.L. (2004). Maintenance of Mouse Male Germ Line Stem Cells In Vitro. Biol. Reprod..

[52] Kokabu S., Gamer L., Cox K., Lowery J., Tsuji K., Raz R., Economides A., Katagiri T., Rosen V. (2012). BMP3 Suppresses Osteoblast Differentiation of Bone Marrow Stromal Cells via Interaction with Acvr2b. Mol. Endocrinol..

[53] Hofmann M.C. (2008). Gdnf Signaling Pathways within the Mammalian Spermatogonial Stem Cell Niche. Mol. Cell. Endocrinol..

[54] Meng X., Lindahl M., Hyvönen M.E., Parvinen M., De Rooij D.G., Hess M.W., Raatikainen-Ahokas A., Sainio K., Rauvala H., Lakso M. (2000). Regulation of cell fate decision of undifferentiated spermatogonia by GDNF. Science.

[55] Costa G.M.J., Avelar G.F., Rezende-Neto J.V., Campos-Junior P.H.A., Lacerda S.M.S.N., Andrade B.S.C., Thomé R.G., Hofmann M.C., Franca L.R. (2012). Spermatogonial Stem Cell Markers and Niche in Equids. PLoS ONE.

[56] Oatley J.M., Oatley M.J., Avarbock M.R., Tobias J.W., Brinster R.L. (2009). Colony stimulating factor 1 is an extrinsic stimulator of mouse spermatogonial stem cell self-renewal. Development.

[57] Hamra F.K., Chapman K.M., Nguyen D.M., Williams-Stephens A.A., Hammer R.E., Garbers D.L. (2005). Self renewal, expansion, and transfection of rat spermatogonial stem cells in culture. Proc. Natl. Acad. Sci. USA.

[58] Li Y., Zhang Y., Zhang X., Sun J., Hao J. (2014). BMP4/Smad signaling pathway induces the differentiation of mouse spermatogonial stem cells via upregulation of Sohlh2. Anat. Rec..

[59] Yang Y., Feng Y., Feng X., Liao S., Wang X., Gan H., Wang L., Lin X., Han C. (2016). BMP4 Cooperates with Retinoic Acid to Induce the Expression of Differentiation Markers in Cultured Mouse Spermatogonia. Stem Cells Int..

[60] Garcia T.X., Hofmann M.C. (2015). Regulation of germ line stem cell homeostasis. Anim. Reprod..

[61] Jing S., Wen D., Yu Y., Holst P.L., Luo Y., Fang M., Tamir R., Antonio L., Hu Z., Cupples R. (1996). GDNF-induced activation of the Ret protein tyrosine kinase is mediated by GDNFR-α, a novel receptor for GDNF. Cell.

[62] Kawase E., Wong M.D., Ding B.C., Xie T. (2004). Gbb/Bmp signaling is essential for maintaining germline stem cells and for repressing bam transcription in the Drosophila testis. Development.

[63] Skryabin G.O., Komelkov A.V., Savelyeva E.E., Tchevkina E.M. (2020). Lipid Rafts in Exosome Biogenesis. Biochemistry.

[64] Tan S.S., Yin Y., Lee T., Lai R.C., Yeo R.W.Y., Zhang B., Choo A., Lim S.K. (2013). Therapeutic MSC exosomes are derived from lipid raft microdomains in the plasma membrane. J. Extracell. Vesicles.

[65] Evans IV W.E., Coyer R.L., Sandusky M.F., Van Fleet M.J., Moore J.G., Nyquist S.E. (2003). Characterization of membrane rafts isolated from rat sertoli cell cultures: Caveolin and flotillin-1 content. J. Androl..

[66] Wei D., Zhan W., Gao Y., Huang L., Gong R., Wang W., Zhang R., Wu Y., Gao S., Kang T. (2020). RAB31 marks and controls an ESCRT-independent exosome pathway. Cell Res..

[67] Essandoh K., Yang L., Wang X., Huang W., Qin D., Hao J., Wang Y., Zingarelli B., Peng T., Fan G.C. (2015). Blockade of exosome generation with GW4869 dampens the sepsis-induced inflammation and cardiac dysfunction. Biochim. Biophys. Acta-Mol. Basis Dis..

[68] Wang X., Huang W., Liu G., Cai W., Millard R.W., Wang Y., Chang J., Peng T., Fan G.C. (2014). Cardiomyocytes mediate anti-angiogenesis in type 2 diabetic rats through the exosomal transfer of miR-320 into endothelial cells. J. Mol. Cell. Cardiol..

[69] Kosaka N., Iguchi H., Yoshioka Y., Takeshita F., Matsuki Y., Ochiya T. (2010). Secretory mechanisms and intercellular transfer of microRNAs in living cells. J. Biol. Chem..

[70] Pramod R.K., Mitra A. (2014). In vitro culture and characterization of spermatogonial stem cells on Sertoli cell feeder layer in goat (*Capra hircus*). J. Assist. Reprod. Genet..

[71] Chen L.-Y., Brown P.R., Willis W.B., Eddy E.M. (2014). Peritubular myoid cells participate in male mouse spermatogonial stem cell maintenance. Endocrinology.

[72] Yoshida S., Sukeno M., Nabeshima Y.I. (2007). A vasculature-associated niche for undifferentiated spermatogonia in the mouse testis. Science.

[73] Chiarini-Garcia H., Hornick J.R., Griswold M.D., Russell L.D. (2001). Distribution of type A spermatogonia in the mouse is not random. Biol. Reprod..

[74] Chan F., Oatley M.J., Kaucher A.V., Yang Q.-E., Bieberich C.J., Shashikant C.S., Oatley J.M. (2014). Functional and molecular features of the Id4+ germline stem cell population in mouse testes. Genes Dev..

[75] Sakib S., Voigt A., de Lima e Martins Lara N., Su L., Ungrin M., Rancourt D., Dobrinski I. (2021). The Proliferation of Pre-Pubertal Porcine Spermatogonia in Stirred Suspension Bioreactors Is Partially Mediated by the Wnt/β-Catenin Pathway. Int. J. Mol. Sci..

[76] Voigt A.L., Kondro D.A., Powell D., Valli-Pulaski H., Ungrin M., Stukenborg J.B., Klein C., Lewis I.A., Orwig K.E., Dobrinski I. (2021). Unique metabolic phenotype and its transition during maturation of juvenile male germ cells. FASEB J..

[77] Matsui Y., Zsebo K., Hogan B.L.M. (1992). Derivation of pluripotential embryonic stem cells from murine primordial germ cells in culture. Cell.

[78] Resnick J.L., Bixler L.S., Cheng L., Donovan P.J. (1992). Long-term proliferation of mouse primordial germ cells in culture. Nature.

[79] Spradling A., Drummond-Barbosa D., Kai T. (2001). Stem cells find their niche. Nature.

[80] Discher D.E., Mooney D.J., Zandstra P.W. (2009). Growth factors, matrices, and forces combine and control stem cells. Science.

[81] Guilak F., Cohen D.M., Estes B.T., Gimble J.M., Liedtke W., Chen C.S. (2009). Control of stem cell fate by physical interactions with the extracellular matrix. Cell Stem Cell.

[82] Wang J.H.C., Thampatty B.P. (2008). Mechanobiology of adult and stem cells. Int. Rev. Cell Mol. Biol..

[83] Geiger B., Spatz J.P., Bershadsky A.D. (2009). Environmental sensing through focal adhesions. Nat. Rev. Mol. Cell Biol..

[84] Schwartz M.A., DeSimone D.W. (2008). Cell adhesion receptors in mechanotransduction. Curr. Opin. Cell Biol..

[85] Park J.E., Park M.H., Kim M.S., Park Y.R., Yun J.I., Cheong H.T., Kim M., Choi J.H., Lee E., Lee S.T. (2017). Porcine spermatogonial stem cells self-renew effectively in a three dimensional culture microenvironment. Cell Biol. Int..

[86] Zhao X., Wan W., Li B., Zhang X., Zhang M., Wu Z., Yang H. (2022). Isolation and in vitro expansion of porcine spermatogonial stem cells. Reprod. Domest. Anim..

[87] Yang Y., Lin Q., Zhou C., Li Q., Li Z., Cao Z., Liang J., Li H., Mei J., Zhang Q. (2020). A Testis-Derived Hydrogel as an Efficient Feeder-Free Culture Platform to Promote Mouse Spermatogonial Stem Cell Proliferation and Differentiation. Front. Cell Dev. Biol..

[88] Faught E., Henrickson L., Vijayan M.M. (2017). Plasma exosomes are enriched in Hsp70 and modulated by stress and cortisol in rainbow trout. J. Endocrinol..

[89] De Jong O.G., Verhaar M.C., Chen Y., Vader P., Gremmels H., Posthuma G., Schiffelers R.M., Gucek M., van Balkom B.W.M. (2012). Cellular stress conditions are reflected in the protein and RNA content of endothelial cell-derived exosomes. J. Extracell. Vesicles.

[90] Zou W., Lai M., Zhang Y., Zheng L., Xing Z., Li T., Zou Z., Song Q., Zhao X., Xia L. (2019). Exosome Release Is Regulated by mTORC1. Adv. Sci..

[91] Baskaran S., Panner Selvam M.K., Agarwal A. (2020). Exosomes of male reproduction. Adv. Clin. Chem..

[92] Li Q., Li H., Liang J., Mei J., Cao Z., Zhang L., Luo J., Tang Y., Huang R., Xia H. (2021). Sertoli cell-derived exosomal MicroRNA-486-5p regulates differentiation of spermatogonial stem cell through PTEN in mice. J. Cell. Mol. Med..

[93] Menck K., Sönmezer C., Worst T.S., Schulz M., Dihazi G.H., Streit F., Erdmann G., Kling S., Boutros M., Binder C. (2017). Neutral sphingomyelinases control extracellular vesicles budding from the plasma membrane. J. Extracell. Vesicles.

[94] Tang L., Bondareva A., González R., Rodriguez-Sosa J.R., Carlson D.F., Webster D., Fahrenkrug S., Dobrinski I. (2018). TALEN-mediated gene targeting in porcine spermatogonia. Mol. Reprod. Dev..

[95] Lou J., Megee S., Dobrinski I. (2009). Asymmetric distribution of UCH-L1 in spermatogonia is associated with maintenance and differentiation of spermatogonial stem cells. J. Cell. Physiol..

[96] Théry C., Amigorena S., Raposo G., Clayton A. (2006). Isolation and Characterization of Exosomes from Cell Culture Supernatants and Biological Fluids. Curr. Protoc. Cell Biol..

