# Supplementary material for: A Role for Exchange of Extracellular Vesicles in Porcine Spermatogonial Co-Culture
Source: Int J Mol Sci. 2022 Apr 20;23(9):4535. doi: 10.3390/ijms23094535 (PMC9103065; doi:10.3390/ijms23094535)
Supplement: Supplementary file 1 [file ijms-23-04535-s001.zip › ijms-1673865-supplementary.pdf]

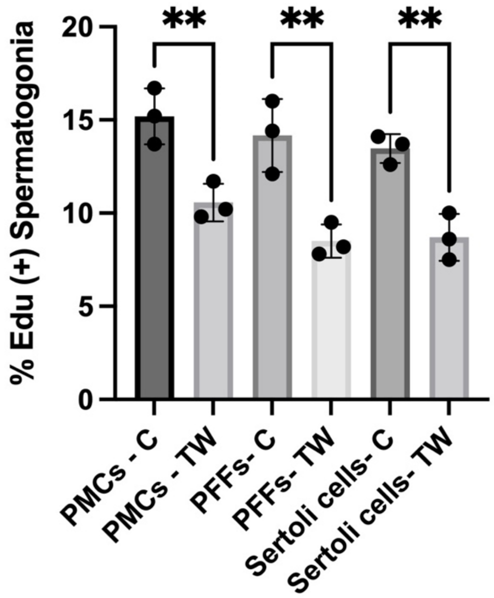

Figure S1: Proliferation of spermatogonia co-cultured in contact with feeders is higher than proliferation of spermatogonia co-cultured with feeders with physical separation using transwell inserts. Percent of UCH-L1 (+) spermatogonia from 1- week-old pigs that are also EdU (+), in co-culture conditions at 1 week of culture, using PMC, PFF, and Sertoli cell feeders. n = 3, mean ± SD. ** $p \leq 0.01$.

Table S1: Reagents and Resources.

REAGENT or RESOURCE	SOURCE	IDENTIFIER
Antibodies		
Rabbit monoclonal IgG anti PGP 9.5	Abcam	CAT#108986
Mouse monoclonal IgG2a anti PGP-9.5	Abcam	CAT#8189
Mouse monoclonal IgG2a anti Gata-4	Santa Cruz Biotechnology	CAT#sc-25310
Rabbit polyclonal IgG2a anti desmin	Abcam	CAT#ab8592
Mouse monoclonal biotinylated- anti CD-31	Abcam	CAT#ab199734
Alexa Flour 488 Donkey Anti-Mouse IgG	Thermo Fisher	CAT#A21202
Alexa Flour 594 Donkey Anti-Rabbit IgG	Thermo Fisher	CAT#A21207
Alexa Flour 594 Donkey Anti-Mouse IgG	Thermo Fisher	CAT#A21203
Biological Samples		
1-week-old pig testis tissue	Sunterra Farms Ltd,Acme,AB, Canada	N/A
8-week-old pig testis tissue	University of Alberta, Edmonton, AB, Canada	N/A
Chemicals, Peptides, and Recombinant Proteins		
GDNF Recombinant human	Bio Technology	CAT#212-Gd-010
EGF Recombinant human	Bio Technology	CAT#236EG200
GFRA	Bio Technology	CAT#560GR100
Insulin	Sigma	CAT#I0516
Holo-Transferrin	Sigma	CAT#T12843
Beta-Mercaptoethanol	Sigma	CAT#M3148
Bovine serum albumin (BSA)	Sigma	CAT#A7906
GW4869	Sigma	CAT# D1692-5MG
PKH67 Green Fluorescent Cell Linker Kit for General Cell Membrane Labeling	Sigma	CAT# PKH67GL-1KT

CAS block	ThermoFisher	CAT#008120
Mitomycin- C	Sigma	CAT#M4287-2MG
Critical Commercial Assays		
RNeasy Mini Kit	Quiagen	CAT#74104
SSoFast EvaGreen Supermix with Low ROX	Bio-Rad	CAT#172-5211
Click-iT™ EdU Imaging Kit with Alexa Fluor™ 488	Thermo Fisher	CAT#C10086
Click-iT™ TUNEL Alexa Fluor™ 488 Imaging Assay, for microscopy & HSC	Thermo Fisher	CAT#C10245
Vybrant™ Alexa Fluor™ 488 Lipid Raft Labeling Kit	ThermoFisher	CAT#V34403
Oligonucleotides		
See Supplemental Table S2		
Software and Algorithms		
ImageJ	N/A	https://imagej.nih.gov/ij/
GraphPad Prism Version 9.3.1	N/A	https://www.graphpad.com/scientific-software/prism/

Table S2: Oligonucleotides.

GDNF	*Sus scrofa* glial cell derived neurotrophic factor	F: TGTCGTGGCTGTCTGCCTGGTG R: GGCGCCTCGGGAGGCCTCTTA	Kakiuchi et al., 2018
BMP3	*Sus scrofa* bone morphogenetic protein 3	F:TCTCCCCCAAGTCCTTCGAT R:ACCCCCACAGCTCTGACTAT	NM_001206388.1
BMP4	*Sus scrofa* bone morphogenetic protein 4	F:AGCTTTCACCACGAAGAACA R:GGTGGGGGCTTCATAACCTC	NM_001101031.2
VEGF	*Sus scrofa* vascular endothelial growth factor A (VEGFA), mRNA	F: ATCGCCATGCAGATTATGCG R: AAATGCTTTCTCCGCTCCGA	NM_214084.1
KIT	*Sus scrofa* KIT proto-oncogene, receptor tyrosine kinase	F:GTGGTCAAAGGAAACGCTCG R:CCCATAGGACCAGACATCGC	NM_001044525.1
NELL2	*Sus scrofa* EGFL like 2	F: CAAAACGTCAGCCAAGCTGT R:TCCATTCAGGCATGTGCAGT	XM_021092577.1
IGF1	*Sus scrofa* insulin like	F:GCCCAAGGCTCAGAAGGAAGTA	NM_214256.1

	growth factor 1	R:TAACTCGTGCAGAGCAAAGGA	
EGF	*Sus scrofa* epidermal growth factor (EGF)	F:ACGGATTTGCCCTGACCCTA<br>R:AGTCTCTGTGCTGACATCGC	NM_214020.2
FGF-2	*Sus scrofa* fibroblast growth factor 2	F:CGGTTGTACTGCAAAAACGG<br>R: TGAAGTTGTAGTTTGATGTGAGGG	XM_021100546.1
CSF-1	*Sus scrofa* colony stimulating factor 1	F: ACCATGCGCTTCAGAGACAA<br>R: TGGAGGGGCGTCTCATAGAA	NM_001244523.1

Table S3: Transwell Experiment Conditions.

Condition	Description
Contact (C)	Spermatogonia seeded directly on top of feeders
Transwell (TW)	Feeders placed on the bottom of wells, and spermatogonia in the transwell inserts
Co-culture- conditioned media (CCCM)	Feeder-free culture using conditioned

	media from co-cultures
Feeder- conditioned media (FCM)	Feeder-free culture using conditioned media from feeders
Spermatogonia on Co-cultures (Spermatogonia on CC)	Spermatogonia and feeders placed on the bottom of wells, and a second group of spermatogonia placed in the transwell inserts (1.0 x 10^5 spermatogonia in the insert and 1.0 x 10^5 spermatogonia in contact with the feeder cells)
Spermatogonia in transwells (SG in TW)	Spermatogonia cultured feeder-free in the transwell inserts in unconditioned α-MEM with additives
Spermatogonia under transwells (SG under TW)	Spermatogonia cultured feeder-free in unconditioned α-MEM with additives in the bottom of the well, with an empty transwell insert above

Table S4: Feeder Cell Gene Expression Data.

Gene	ΔCT values			Mean FC			p- value		
	Sertoli cells	PMCs	PFFs	Sertoli cells	PMCs	PFFs	SC-PMC	SC-PFF	PMC-PFF

IGF1	5.537	4.326	5.18	1	2.794	1.961	0.0674	0.6887	0.3612
EGF	10.43	9.714	8.900	1	1.765	3.068	0.2719	0.1079	0.2820
BMP4	1.973	3.347	4.059	1	0.392	0.2560	0.0081	0.0231	0.2161
BMP3	10.08	9.533	5.836	1	2.341	21.229	0.5403	0.0069	0.0071
NELL2	5.336	7.684	8.573	1	0.265	0.1120	0.0345	0.0149	0.2367
TNF	12.13	10.17	11.12	1	8.320	2.074	0.2000	0.4032	0.4000
FGF2	2.416	1.948	1.808	1	1.717	1.558	0.5510	0.1404	0.8468
CSF1	1.714	1.94	0.5123	1	1.272	2.427	0.8096	0.1086	0.1802
GDNF	3.048	5.434	6.010	1	0.217	0.1510	0.0092	0.0058	0.1989
KIT	3.688	4.414	7.028	1	0.648	0.1070	0.1952	0.0176	0.0544
VEGF	2.504	1.398	2.147	1	2.818	2.132	>0.9999	0.7000	0.3559
HPRT	3.029	3.192	3.951	1	0.9360	0.5286	0.5998	0.0055	0.0839
EIF	0.5653	0.4297	0.2737	1	1.099	1.230	0.1000	0.0903	0.4000
RLP	−3.594	−3.620	−4.225	1	1.056	1.550	0.9191	0.0070	0.1158
